# Di-μ-chromato-κ^4^ *O*:*O*′-bis­[bis­(phenan­throline-κ^2^ *N*,*N*′)cadmium(II)] dihydrate

**DOI:** 10.1107/S160053680900823X

**Published:** 2009-03-14

**Authors:** Hai-Xing Liu, Fang-Fang Jian, Jing Wang

**Affiliations:** a Microscale Science Institute, Weifang University, Weifang 261061, People’s Republic of China; b New Materials and Function Coordination Chemistry Laboratory, Qingdao University of Science and Technology, Qingdao 266042, People’s Republic of China

## Abstract

In the title compound, [Cd_2_Cr_2_O_8_(C_12_H_8_N_2_)_4_]·2H_2_O, which was obtained by hydro­thermal reaction of CdCO_3_ and phenanthroline with K_2_CrO_4_ at 393 K, two distorted Cd(N_4_O_2_) octa­hedra are linked through μ_2_-bridging chromate anions, forming a centrosymmetric tetra­nuclear eight-membered ring complex. The water mol­ecules link the chromate O atoms *via* inter­molecular O—H⋯O hydrogen bonds. These aggregates pack to a three-dimensional network through weak inter­molecular C—H⋯O and C—H⋯π hydrogen-bonding contacts.

## Related literature

For the properties of multimetallic complexes, see: Costisor *et al.* (2001 ▶). For the structures of heterometallic macrocyclic rings, see: Larsen *et al.* (2003 ▶); Timco *et al.* (2005 ▶). For related structures, see: Dai *et al.* (2002 ▶); Chaudhuri *et al.* (1988 ▶); Yoshikawa *et al.* (2002 ▶).
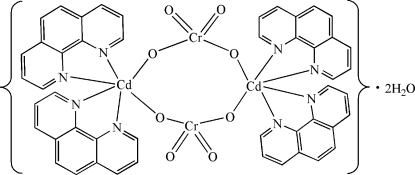


## Experimental

### 

#### Crystal data

[Cd_2_Cr_2_O_8_(C_12_H_8_N_2_)_4_]·2H_2_O
*M* *_r_* = 1213.65
Monoclinic, 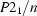

*a* = 11.2303 (13) Å
*b* = 13.6892 (16) Å
*c* = 14.5352 (19) Å
β = 91.928 (1)°
*V* = 2233.3 (5) Å^3^
*Z* = 2
Mo *K*α radiation
μ = 1.48 mm^−1^
*T* = 298 K
0.13 × 0.08 × 0.05 mm

#### Data collection

Bruker SMART CCD area-detector diffractometer
Absorption correction: multi-scan (*SADABS*; Bruker, 1997 ▶) *T*_min_ = 0.830, *T*_max_ = 0.930
11590 measured reflections
3922 independent reflections
2145 reflections with *I* > 2σ(*I*)
*R*_int_ = 0.096

#### Refinement

*R*[*F* ^2^ > 2σ(*F* ^2^)] = 0.045
*wR*(*F* ^2^) = 0.065
*S* = 0.86
3922 reflections
316 parameters
H-atom parameters constrained
Δρ_max_ = 0.50 e Å^−3^
Δρ_min_ = −0.52 e Å^−3^

### 

Data collection: *SMART* (Bruker, 1997 ▶); cell refinement: *SAINT* (Bruker, 1997 ▶); data reduction: *SAINT*; program(s) used to solve structure: *SHELXS97* (Sheldrick, 2008 ▶); program(s) used to refine structure: *SHELXL97* (Sheldrick, 2008 ▶); molecular graphics: *SHELXTL* (Sheldrick, 2008 ▶); software used to prepare material for publication: *SHELXTL*.

## Supplementary Material

Crystal structure: contains datablocks global, I. DOI: 10.1107/S160053680900823X/si2157sup1.cif

Structure factors: contains datablocks I. DOI: 10.1107/S160053680900823X/si2157Isup2.hkl

Additional supplementary materials:  crystallographic information; 3D view; checkCIF report

## Figures and Tables

**Table 1:** Selected geometric parameters (Å, °)

Cd1—O2	2.215 (4)
Cd1—O1	2.226 (4)
Cd1—N2	2.370 (5)
Cd1—N1	2.376 (5)
Cd1—N4	2.394 (5)
Cd1—N3	2.397 (5)
O1—Cr1	1.660 (4)
O2—Cr1^i^	1.683 (4)
O3—Cr1	1.638 (4)
O4—Cr1	1.619 (4)

Symmetry code: (i) 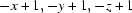.

**Table 2:** Hydrogen-bond geometry (Å, °)

*D*—H⋯*A*	*D*—H	H⋯*A*	*D*⋯*A*	*D*—H⋯*A*
O5—H5*A*⋯O2^ii^	0.85	2.13	2.849 (6)	142
O5—H5*B*⋯O4^iii^	0.85	2.40	3.122 (6)	144
C2—H2⋯O3^iv^	0.93	2.49	3.274 (7)	142
C3—H3⋯O3^iii^	0.93	2.50	3.352 (8)	153
C9—H9⋯O3^v^	0.93	2.48	3.391 (7)	168
C10—H10⋯O3	0.93	2.55	3.478 (7)	175
C12—H12⋯O4^ii^	0.93	2.58	3.423 (7)	151
C20—H20⋯O5^vi^	0.93	2.49	3.344 (8)	152
C23—H23⋯*Cg*1^vii^	0.93	2.61	3.509 (7)	164

Symmetry codes: (ii) 
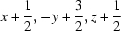
; (iii) 
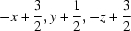
; (iv) 
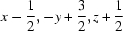
; (v) 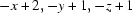; (vi) 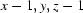; (vii) 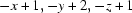. *Cg*1 is the centroid of atoms N1,C1–C5.

**Fig. 1.**
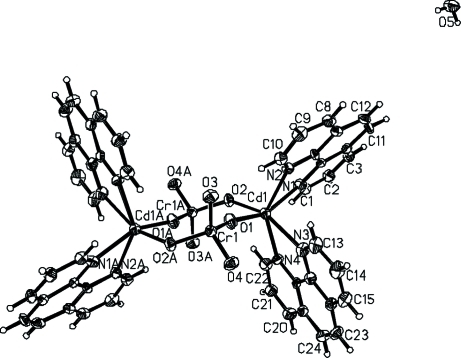
The molecular structure of the title compound with the atom-labeling scheme. Displacement ellipsoids are drawn at the 30% probability level.

**Fig. 2.**
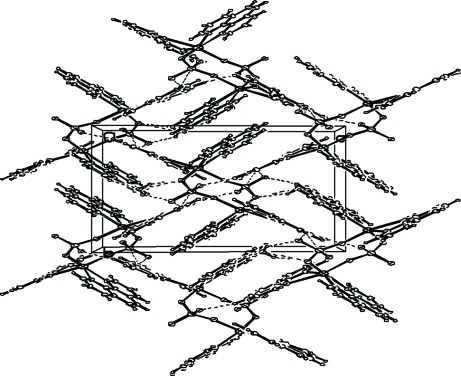
The packing view of the molecules of (I) along the crystallographic a direction.

## supplementary crystallographic information

### Comment

In recent decades, research on multimetallic complexes has grown in modern inorganic chemistry, because of searching for new materials, exhibiting exciting magnetic properties, electrical and optical properties (Costisor *et al.*, 2001). But the heterometallic systems are rare because of the difficult synthesis. In contrast to the heterometallic macrocylic ring structures reported (Larsen *et al.*, 2003 & Timco *et al.*, 2005), we describe the synthesis and structure of the title compound, which represents a centrosymmetric heterobinuclear eight-membered ring system.

The title structure (Fig. 1) has a centrosymmetric eight-membered ring, build up of [Cd(phenanthroline)_2_]^2+^, [CrO_4_]^2-^ units and two free water molecules. Each Cd atom is coordinated with four N atoms from phenanthroline ligands and two O atoms, presenting a distorted octahedral geometry. The Cr atoms are tetrahedrally coordinated. Two distorted Cd(N_4_O_2_) octahedra are linked through bridging chromate anions to form the centrosymmetric tetranuclear eight-membered ring complex. The mean Cd—O, Cr—O and Cd—N bond lengths are similar to the values reported (Dai *et al.*, 2002, Chaudhuri *et al.*, 1988, Yoshikawa *et al.*, 2002). The Cr1^i^—O2—Cd1, O1—Cr1—O2, O2—Cd1—O1 angles are 133.1 (2)°, 109.40 (18)°, and 97.47 (13)°, respectively. Other selected geometrical parameters are given in Table 1. The dihedral angle between the phenanthroline ligands is 89.00 (1)°. The free water molecules link the chromate oxygen atoms *via* intermolecular O—H···O hydrogen bonds. The intermolecular C—H···O hydrogen bonds and the C—H···π interactions (Table 2) cause the crystal packing to be energetically preferable and generate a three-dimensional network as shown in Fig. 2.

### Experimental

All commercially obtained reagent-grade chemicals were used without further purication. CdCO_3_ (3.40 g, 2.00 mmol) was dissolved in water and methanol (2:1 *v*/*v*, 30 ml), mixed with phenanthroline (6.00 g, 3.00 mmol). After stirring for 0.5 h, K_2_CrO_4_ (1.94 g, 1.00 mmol) was added to the mixture. The hydrothermal reaction was conducted at 393 K for 4 h. The yellow prism crystals were collected, after cooling and filtering (yield 1.20 g). Analysis calculated for C_48_H_36_Cd_2_Cr_2_N_8_O_10_: C 47.46, H 2.97, N 9.22%; found: C 47.44, H 3.03, N 9.20%.

### Refinement

H atoms were positioned geometrically and allowed to ride on their parent atoms, with N—H and C—H distances of 0.86 and 0.93–0.96 Å, respectively, and with *U*_iso_(H) = 1.2*U*_eq_ of the parent atoms.

### Crystal data

**Table:**

[Cd_2_Cr_2_O_8_(C_12_H_8_N_2_)_4_]·2H_2_O	*F*(000) = 1208
*M**_r_* = 1213.65	*D*_x_ = 1.805 Mg m^−^^3^
Monoclinic, *P*2_1_/*n*	Mo *K*α radiation, λ = 0.71073 Å
*a* = 11.2303 (13) Å	Cell parameters from 1518 reflections
*b* = 13.6892 (16) Å	θ = 2.3–25.0°
*c* = 14.5352 (19) Å	µ = 1.48 mm^−^^1^
β = 91.928 (1)°	*T* = 298 K
*V* = 2233.3 (5) Å^3^	Prism, yellow
*Z* = 2	0.13 × 0.08 × 0.05 mm

### Data collection

**Table:**

Bruker SMART CCD area-detector diffractometer	3922 independent reflections
Radiation source: fine-focus sealed tube	2145 reflections with *I* > 2σ(*I*)
graphite	*R*_int_ = 0.096
φ and ω scans	θ_max_ = 25.0°, θ_min_ = 2.0°
Absorption correction: multi-scan (*SADABS*; Bruker, 1997)	*h* = −13→9
*T*_min_ = 0.830, *T*_max_ = 0.930	*k* = −16→14
11590 measured reflections	*l* = −17→17

### Refinement

**Table:**

Refinement on *F*^2^	Primary atom site location: structure-invariant direct methods
Least-squares matrix: full	Secondary atom site location: difference Fourier map
*R*[*F*^2^ > 2σ(*F*^2^)] = 0.045	Hydrogen site location: inferred from neighbouring sites
*wR*(*F*^2^) = 0.065	H-atom parameters constrained
*S* = 0.86	*w* = 1/[σ^2^(*F*_o_^2^) + (0.0001*P*)^2^] where *P* = (*F*_o_^2^ + 2*F*_c_^2^)/3
3922 reflections	(Δ/σ)_max_ = 0.001
316 parameters	Δρ_max_ = 0.50 e Å^−^^3^
0 restraints	Δρ_min_ = −0.52 e Å^−^^3^

### Special details

**Table:**

Geometry. All e.s.d.'s (except the e.s.d. in the dihedral angle between two l.s. planes) are estimated using the full covariance matrix. The cell e.s.d.'s are taken into account individually in the estimation of e.s.d.'s in distances, angles and torsion angles; correlations between e.s.d.'s in cell parameters are only used when they are defined by crystal symmetry. An approximate (isotropic) treatment of cell e.s.d.'s is used for estimating e.s.d.'s involving l.s. planes.
Refinement. Refinement of *F*^2^ against ALL reflections. The weighted *R*-factor *wR* and goodness of fit *S* are based on *F*^2^, conventional *R*-factors *R* are based on *F*, with *F* set to zero for negative *F*^2^. The threshold expression of *F*^2^ > σ(*F*^2^) is used only for calculating *R*-factors(gt) *etc*. and is not relevant to the choice of reflections for refinement. *R*-factors based on *F*^2^ are statistically about twice as large as those based on *F*, and *R*- factors based on ALL data will be even larger.

### Fractional atomic coordinates and isotropic or equivalent isotropic displacement parameters (Å^2^)

**Table:**

	*x*	*y*	*z*	*U*_iso_*/*U*_eq_	
Cd1	0.54305 (4)	0.67292 (3)	0.58619 (3)	0.03350 (14)	
N1	0.5517 (4)	0.7535 (3)	0.7310 (3)	0.0336 (13)	
N2	0.7407 (4)	0.6723 (3)	0.6487 (3)	0.0345 (12)	
N3	0.6012 (5)	0.8015 (3)	0.4851 (4)	0.0439 (15)	
N4	0.3768 (4)	0.7747 (3)	0.5434 (3)	0.0359 (13)	
O1	0.6078 (4)	0.5715 (3)	0.4790 (3)	0.0484 (13)	
O2	0.4197 (3)	0.5701 (3)	0.6521 (3)	0.0407 (12)	
O3	0.7995 (3)	0.4755 (3)	0.4225 (3)	0.0453 (12)	
O4	0.6869 (3)	0.5992 (3)	0.3082 (3)	0.0482 (12)	
O5	0.9994 (4)	1.0021 (3)	1.3274 (3)	0.0759 (16)	
H5A	0.9990	0.9605	1.2840	0.091*	
H5B	0.9505	1.0471	1.3117	0.091*	
Cr1	0.66961 (8)	0.51967 (7)	0.38910 (7)	0.0325 (3)	
C1	0.4611 (5)	0.7872 (4)	0.7770 (4)	0.0391 (17)	
H1	0.3848	0.7689	0.7570	0.047*	
C2	0.4718 (6)	0.8482 (4)	0.8532 (4)	0.0436 (18)	
H2	0.4043	0.8706	0.8819	0.052*	
C3	0.5821 (6)	0.8746 (4)	0.8852 (4)	0.0431 (18)	
H3	0.5907	0.9143	0.9368	0.052*	
C4	0.6834 (5)	0.8415 (4)	0.8399 (4)	0.0341 (15)	
C5	0.6630 (5)	0.7787 (4)	0.7641 (4)	0.0276 (15)	
C6	0.7639 (5)	0.7354 (4)	0.7186 (4)	0.0276 (15)	
C7	0.8814 (6)	0.7601 (4)	0.7509 (4)	0.0346 (16)	
C8	0.9753 (6)	0.7126 (4)	0.7098 (4)	0.0429 (18)	
H8	1.0533	0.7264	0.7289	0.051*	
C9	0.9533 (6)	0.6458 (4)	0.6413 (5)	0.049 (2)	
H9	1.0154	0.6119	0.6152	0.059*	
C10	0.8346 (6)	0.6298 (4)	0.6116 (4)	0.0391 (17)	
H10	0.8206	0.5869	0.5629	0.047*	
C11	0.8043 (6)	0.8687 (4)	0.8675 (4)	0.0435 (18)	
H11	0.8177	0.9134	0.9149	0.052*	
C12	0.8964 (5)	0.8294 (5)	0.8249 (4)	0.0423 (16)	
H12	0.9732	0.8475	0.8437	0.051*	
C13	0.7115 (6)	0.8143 (5)	0.4542 (4)	0.053 (2)	
H13	0.7723	0.7738	0.4766	0.063*	
C14	0.7391 (7)	0.8865 (5)	0.3892 (5)	0.060 (2)	
H14	0.8168	0.8932	0.3700	0.072*	
C15	0.6527 (6)	0.9458 (5)	0.3549 (5)	0.052 (2)	
H15	0.6705	0.9934	0.3119	0.063*	
C16	0.5359 (6)	0.9354 (5)	0.3845 (4)	0.0423 (18)	
C17	0.5154 (6)	0.8613 (4)	0.4499 (4)	0.0380 (17)	
C18	0.3954 (5)	0.8468 (4)	0.4803 (4)	0.0306 (15)	
C19	0.3029 (6)	0.9051 (4)	0.4452 (4)	0.0396 (18)	
C20	0.1869 (6)	0.8889 (5)	0.4757 (4)	0.050 (2)	
H20	0.1234	0.9270	0.4540	0.060*	
C21	0.1692 (5)	0.8152 (5)	0.5387 (5)	0.0480 (18)	
H21	0.0935	0.8025	0.5598	0.058*	
C22	0.2666 (6)	0.7604 (5)	0.5700 (4)	0.0460 (19)	
H22	0.2535	0.7107	0.6122	0.055*	
C23	0.4397 (7)	0.9928 (5)	0.3503 (5)	0.054 (2)	
H23	0.4540	1.0412	0.3071	0.065*	
C24	0.3285 (6)	0.9795 (5)	0.3783 (4)	0.053 (2)	
H24	0.2671	1.0185	0.3544	0.064*	

### Atomic displacement parameters (Å^2^)

**Table:**

	*U*^11^	*U*^22^	*U*^33^	*U*^12^	*U*^13^	*U*^23^
Cd1	0.0289 (2)	0.0347 (3)	0.0369 (3)	0.0001 (3)	0.0019 (2)	−0.0027 (3)
N1	0.021 (3)	0.046 (3)	0.034 (3)	0.003 (3)	0.003 (3)	−0.002 (3)
N2	0.037 (3)	0.032 (3)	0.034 (3)	0.011 (3)	0.002 (3)	−0.008 (3)
N3	0.039 (3)	0.041 (4)	0.053 (4)	0.005 (3)	0.011 (3)	−0.003 (3)
N4	0.035 (3)	0.034 (3)	0.039 (4)	0.002 (3)	0.000 (3)	0.008 (3)
O1	0.044 (3)	0.051 (3)	0.050 (3)	−0.002 (2)	0.007 (2)	−0.018 (2)
O2	0.043 (3)	0.037 (3)	0.043 (3)	−0.015 (2)	0.003 (2)	−0.005 (2)
O3	0.027 (3)	0.052 (3)	0.057 (3)	0.010 (2)	0.003 (2)	0.004 (2)
O4	0.042 (3)	0.049 (3)	0.054 (3)	−0.004 (2)	0.003 (2)	0.019 (2)
O5	0.079 (4)	0.088 (4)	0.059 (4)	0.000 (3)	−0.019 (3)	0.011 (3)
Cr1	0.0274 (6)	0.0340 (6)	0.0361 (7)	−0.0011 (5)	0.0021 (5)	0.0003 (5)
C1	0.030 (4)	0.039 (4)	0.048 (5)	0.000 (3)	0.005 (4)	0.005 (3)
C2	0.044 (4)	0.042 (5)	0.046 (5)	0.009 (4)	0.006 (4)	−0.008 (3)
C3	0.059 (5)	0.037 (4)	0.033 (4)	0.008 (4)	0.001 (4)	−0.012 (3)
C4	0.044 (4)	0.025 (4)	0.033 (4)	0.002 (3)	−0.001 (3)	0.002 (3)
C5	0.034 (4)	0.021 (3)	0.028 (4)	0.002 (3)	−0.002 (3)	0.002 (3)
C6	0.026 (4)	0.030 (4)	0.027 (4)	−0.008 (3)	0.002 (3)	−0.004 (3)
C7	0.032 (4)	0.039 (4)	0.033 (4)	0.001 (3)	−0.002 (3)	0.002 (3)
C8	0.029 (4)	0.053 (5)	0.047 (5)	−0.005 (4)	−0.001 (4)	0.012 (4)
C9	0.035 (4)	0.052 (5)	0.061 (5)	0.007 (4)	0.017 (4)	0.009 (4)
C10	0.041 (4)	0.044 (4)	0.033 (4)	−0.001 (4)	0.009 (4)	−0.001 (3)
C11	0.050 (5)	0.043 (4)	0.037 (4)	−0.017 (4)	−0.009 (4)	−0.008 (3)
C12	0.033 (4)	0.051 (4)	0.042 (4)	−0.008 (4)	−0.010 (3)	0.011 (4)
C13	0.048 (5)	0.045 (5)	0.067 (5)	0.003 (4)	0.017 (4)	−0.004 (4)
C14	0.051 (5)	0.064 (6)	0.067 (6)	−0.017 (5)	0.023 (5)	0.009 (4)
C15	0.063 (5)	0.039 (5)	0.055 (5)	0.003 (4)	0.014 (5)	0.004 (4)
C16	0.057 (5)	0.038 (4)	0.033 (4)	−0.008 (4)	0.009 (4)	0.001 (3)
C17	0.038 (4)	0.036 (4)	0.039 (4)	0.003 (4)	0.005 (4)	−0.006 (3)
C18	0.037 (4)	0.022 (4)	0.033 (4)	−0.004 (3)	0.001 (3)	−0.003 (3)
C19	0.048 (5)	0.032 (4)	0.039 (5)	−0.003 (4)	0.000 (4)	−0.001 (3)
C20	0.048 (5)	0.049 (5)	0.053 (5)	0.018 (4)	−0.004 (4)	−0.005 (4)
C21	0.032 (4)	0.049 (5)	0.063 (5)	0.002 (4)	−0.005 (4)	0.007 (4)
C22	0.046 (5)	0.052 (5)	0.040 (5)	−0.004 (4)	0.008 (4)	0.003 (4)
C23	0.075 (6)	0.040 (5)	0.049 (5)	−0.005 (5)	0.008 (5)	0.015 (4)
C24	0.060 (5)	0.050 (5)	0.048 (5)	0.004 (4)	−0.006 (4)	0.009 (4)

### Geometric parameters (Å, °)

**Table:**

Cd1—O2	2.215 (4)	C7—C8	1.390 (7)
Cd1—O1	2.226 (4)	C7—C12	1.440 (8)
Cd1—N2	2.370 (5)	C8—C9	1.367 (8)
Cd1—N1	2.376 (5)	C8—H8	0.9300
Cd1—N4	2.394 (5)	C9—C10	1.405 (8)
Cd1—N3	2.397 (5)	C9—H9	0.9300
N1—C1	1.319 (6)	C10—H10	0.9300
N1—C5	1.369 (7)	C11—C12	1.336 (7)
N2—C10	1.334 (6)	C11—H11	0.9300
N2—C6	1.353 (6)	C12—H12	0.9300
N3—C13	1.344 (7)	C13—C14	1.408 (8)
N3—C17	1.352 (7)	C13—H13	0.9300
N4—C22	1.324 (7)	C14—C15	1.348 (8)
N4—C18	1.369 (6)	C14—H14	0.9300
O1—Cr1	1.660 (4)	C15—C16	1.401 (8)
O2—Cr1^i^	1.683 (4)	C15—H15	0.9300
O3—Cr1	1.638 (4)	C16—C23	1.412 (9)
O4—Cr1	1.619 (4)	C16—C17	1.415 (8)
O5—H5A	0.8501	C17—C18	1.445 (7)
O5—H5B	0.8500	C18—C19	1.394 (8)
Cr1—O2^i^	1.683 (4)	C19—C20	1.408 (8)
C1—C2	1.388 (7)	C19—C24	1.442 (8)
C1—H1	0.9300	C20—C21	1.381 (7)
C2—C3	1.357 (8)	C20—H20	0.9300
C2—H2	0.9300	C21—C22	1.390 (8)
C3—C4	1.408 (7)	C21—H21	0.9300
C3—H3	0.9300	C22—H22	0.9300
C4—C5	1.410 (7)	C23—C24	1.339 (8)
C4—C11	1.452 (8)	C23—H23	0.9300
C5—C6	1.456 (7)	C24—H24	0.9300
C6—C7	1.426 (8)		
			
O2—Cd1—O1	97.47 (13)	C8—C7—C6	117.1 (6)
O2—Cd1—N2	115.01 (15)	C8—C7—C12	123.9 (6)
O1—Cd1—N2	86.70 (15)	C6—C7—C12	119.0 (5)
O2—Cd1—N1	85.37 (15)	C9—C8—C7	120.3 (6)
O1—Cd1—N1	154.79 (16)	C9—C8—H8	119.9
N2—Cd1—N1	69.64 (15)	C7—C8—H8	119.9
O2—Cd1—N4	89.34 (15)	C8—C9—C10	118.3 (6)
O1—Cd1—N4	116.81 (16)	C8—C9—H9	120.9
N2—Cd1—N4	144.46 (17)	C10—C9—H9	120.9
N1—Cd1—N4	88.19 (16)	N2—C10—C9	124.3 (6)
O2—Cd1—N3	156.75 (17)	N2—C10—H10	117.9
O1—Cd1—N3	85.81 (15)	C9—C10—H10	117.9
N2—Cd1—N3	88.10 (17)	C12—C11—C4	120.0 (6)
N1—Cd1—N3	101.44 (17)	C12—C11—H11	120.0
N4—Cd1—N3	68.90 (17)	C4—C11—H11	120.0
C1—N1—C5	116.4 (5)	C11—C12—C7	122.6 (6)
C1—N1—Cd1	127.0 (4)	C11—C12—H12	118.7
C5—N1—Cd1	116.0 (4)	C7—C12—H12	118.7
C10—N2—C6	116.6 (5)	N3—C13—C14	122.8 (6)
C10—N2—Cd1	126.1 (4)	N3—C13—H13	118.6
C6—N2—Cd1	116.1 (4)	C14—C13—H13	118.6
C13—N3—C17	116.6 (5)	C15—C14—C13	119.9 (7)
C13—N3—Cd1	125.1 (5)	C15—C14—H14	120.0
C17—N3—Cd1	118.1 (4)	C13—C14—H14	120.0
C22—N4—C18	117.9 (5)	C14—C15—C16	119.7 (7)
C22—N4—Cd1	124.5 (4)	C14—C15—H15	120.2
C18—N4—Cd1	117.3 (4)	C16—C15—H15	120.2
Cr1—O1—Cd1	166.5 (2)	C15—C16—C23	123.3 (6)
Cr1^i^—O2—Cd1	133.1 (2)	C15—C16—C17	117.0 (7)
H5A—O5—H5B	107.4	C23—C16—C17	119.7 (6)
O4—Cr1—O3	109.6 (2)	N3—C17—C16	124.0 (6)
O4—Cr1—O1	110.4 (2)	N3—C17—C18	117.5 (6)
O3—Cr1—O1	108.4 (2)	C16—C17—C18	118.5 (6)
O4—Cr1—O2^i^	108.5 (2)	N4—C18—C19	122.0 (5)
O3—Cr1—O2^i^	110.5 (2)	N4—C18—C17	117.9 (6)
O1—Cr1—O2^i^	109.40 (18)	C19—C18—C17	120.1 (6)
N1—C1—C2	124.5 (6)	C18—C19—C20	118.7 (6)
N1—C1—H1	117.7	C18—C19—C24	119.2 (6)
C2—C1—H1	117.7	C20—C19—C24	122.1 (7)
C3—C2—C1	119.1 (6)	C21—C20—C19	118.6 (6)
C3—C2—H2	120.4	C21—C20—H20	120.7
C1—C2—H2	120.4	C19—C20—H20	120.7
C2—C3—C4	119.7 (6)	C20—C21—C22	118.9 (6)
C2—C3—H3	120.1	C20—C21—H21	120.6
C4—C3—H3	120.1	C22—C21—H21	120.6
C3—C4—C5	116.7 (6)	N4—C22—C21	123.9 (6)
C3—C4—C11	123.4 (6)	N4—C22—H22	118.1
C5—C4—C11	119.9 (5)	C21—C22—H22	118.1
N1—C5—C4	123.4 (5)	C24—C23—C16	121.8 (6)
N1—C5—C6	117.0 (5)	C24—C23—H23	119.1
C4—C5—C6	119.6 (6)	C16—C23—H23	119.1
N2—C6—C7	123.4 (5)	C23—C24—C19	120.7 (7)
N2—C6—C5	117.9 (5)	C23—C24—H24	119.7
C7—C6—C5	118.7 (5)	C19—C24—H24	119.7
			
O2—Cd1—N1—C1	−55.4 (5)	C11—C4—C5—N1	−176.7 (5)
O1—Cd1—N1—C1	−153.1 (4)	C3—C4—C5—C6	−175.1 (5)
N2—Cd1—N1—C1	−174.2 (5)	C11—C4—C5—C6	4.8 (9)
N4—Cd1—N1—C1	34.1 (5)	C10—N2—C6—C7	2.1 (8)
N3—Cd1—N1—C1	102.1 (5)	Cd1—N2—C6—C7	−166.0 (5)
O2—Cd1—N1—C5	133.8 (4)	C10—N2—C6—C5	−176.5 (5)
O1—Cd1—N1—C5	36.1 (6)	Cd1—N2—C6—C5	15.3 (6)
N2—Cd1—N1—C5	15.0 (4)	N1—C5—C6—N2	−1.5 (8)
N4—Cd1—N1—C5	−136.7 (4)	C4—C5—C6—N2	177.2 (5)
N3—Cd1—N1—C5	−68.6 (4)	N1—C5—C6—C7	179.8 (5)
O2—Cd1—N2—C10	102.9 (4)	C4—C5—C6—C7	−1.5 (8)
O1—Cd1—N2—C10	6.2 (5)	N2—C6—C7—C8	−2.7 (9)
N1—Cd1—N2—C10	177.3 (5)	C5—C6—C7—C8	175.9 (5)
N4—Cd1—N2—C10	−127.9 (4)	N2—C6—C7—C12	179.1 (5)
N3—Cd1—N2—C10	−79.7 (5)	C5—C6—C7—C12	−2.3 (9)
O2—Cd1—N2—C6	−90.2 (4)	C6—C7—C8—C9	0.2 (9)
O1—Cd1—N2—C6	173.1 (4)	C12—C7—C8—C9	178.3 (6)
N1—Cd1—N2—C6	−15.8 (4)	C7—C8—C9—C10	2.6 (9)
N4—Cd1—N2—C6	38.9 (5)	C6—N2—C10—C9	0.9 (9)
N3—Cd1—N2—C6	87.2 (4)	Cd1—N2—C10—C9	167.7 (5)
O2—Cd1—N3—C13	−156.8 (4)	C8—C9—C10—N2	−3.3 (10)
O1—Cd1—N3—C13	−57.6 (5)	C3—C4—C11—C12	175.7 (6)
N2—Cd1—N3—C13	29.2 (5)	C5—C4—C11—C12	−4.2 (9)
N1—Cd1—N3—C13	98.0 (5)	C4—C11—C12—C7	0.3 (10)
N4—Cd1—N3—C13	−178.5 (5)	C8—C7—C12—C11	−175.1 (6)
O2—Cd1—N3—C17	17.3 (7)	C6—C7—C12—C11	3.0 (10)
O1—Cd1—N3—C17	116.5 (5)	C17—N3—C13—C14	0.7 (10)
N2—Cd1—N3—C17	−156.7 (5)	Cd1—N3—C13—C14	174.9 (5)
N1—Cd1—N3—C17	−87.9 (5)	N3—C13—C14—C15	−0.5 (11)
N4—Cd1—N3—C17	−4.4 (4)	C13—C14—C15—C16	0.2 (11)
O2—Cd1—N4—C22	6.7 (5)	C14—C15—C16—C23	−178.3 (7)
O1—Cd1—N4—C22	104.8 (5)	C14—C15—C16—C17	−0.2 (10)
N2—Cd1—N4—C22	−128.6 (5)	C13—N3—C17—C16	−0.7 (9)
N1—Cd1—N4—C22	−78.7 (5)	Cd1—N3—C17—C16	−175.3 (5)
N3—Cd1—N4—C22	178.3 (5)	C13—N3—C17—C18	178.2 (5)
O2—Cd1—N4—C18	−166.9 (4)	Cd1—N3—C17—C18	3.6 (7)
O1—Cd1—N4—C18	−68.8 (4)	C15—C16—C17—N3	0.4 (10)
N2—Cd1—N4—C18	57.8 (5)	C23—C16—C17—N3	178.6 (6)
N1—Cd1—N4—C18	107.8 (4)	C15—C16—C17—C18	−178.5 (6)
N3—Cd1—N4—C18	4.8 (4)	C23—C16—C17—C18	−0.3 (9)
O2—Cd1—O1—Cr1	168.8 (11)	C22—N4—C18—C19	0.9 (9)
N2—Cd1—O1—Cr1	−76.4 (11)	Cd1—N4—C18—C19	174.9 (4)
N1—Cd1—O1—Cr1	−96.2 (12)	C22—N4—C18—C17	−178.9 (5)
N4—Cd1—O1—Cr1	75.7 (11)	Cd1—N4—C18—C17	−4.9 (7)
N3—Cd1—O1—Cr1	11.9 (11)	N3—C17—C18—N4	0.8 (8)
O1—Cd1—O2—Cr1^i^	−35.6 (3)	C16—C17—C18—N4	179.8 (5)
N2—Cd1—O2—Cr1^i^	−125.5 (3)	N3—C17—C18—C19	−178.9 (6)
N1—Cd1—O2—Cr1^i^	169.6 (3)	C16—C17—C18—C19	0.1 (9)
N4—Cd1—O2—Cr1^i^	81.3 (3)	N4—C18—C19—C20	−0.1 (9)
N3—Cd1—O2—Cr1^i^	61.2 (5)	C17—C18—C19—C20	179.7 (5)
Cd1—O1—Cr1—O4	−24.0 (12)	N4—C18—C19—C24	−179.6 (5)
Cd1—O1—Cr1—O3	96.1 (11)	C17—C18—C19—C24	0.2 (9)
Cd1—O1—Cr1—O2^i^	−143.3 (11)	C18—C19—C20—C21	−0.6 (9)
C5—N1—C1—C2	2.4 (9)	C24—C19—C20—C21	178.9 (6)
Cd1—N1—C1—C2	−168.3 (4)	C19—C20—C21—C22	0.4 (10)
N1—C1—C2—C3	−1.4 (10)	C18—N4—C22—C21	−1.0 (10)
C1—C2—C3—C4	1.2 (10)	Cd1—N4—C22—C21	−174.6 (5)
C2—C3—C4—C5	−2.2 (9)	C20—C21—C22—N4	0.4 (11)
C2—C3—C4—C11	177.9 (6)	C15—C16—C23—C24	178.3 (7)
C1—N1—C5—C4	−3.5 (8)	C17—C16—C23—C24	0.2 (11)
Cd1—N1—C5—C4	168.3 (4)	C16—C23—C24—C19	0.0 (11)
C1—N1—C5—C6	175.1 (5)	C18—C19—C24—C23	−0.2 (10)
Cd1—N1—C5—C6	−13.1 (6)	C20—C19—C24—C23	−179.7 (6)
C3—C4—C5—N1	3.4 (9)		

Symmetry codes: (i) −*x*+1, −*y*+1, −*z*+1.

### Hydrogen-bond geometry (Å, °)

**Table:**

*D*—H···*A*	*D*—H	H···*A*	*D*···*A*	*D*—H···*A*
O5—H5A···O2^ii^	0.85	2.13	2.849 (6)	142
O5—H5B···O4^iii^	0.85	2.40	3.122 (6)	144
C2—H2···O3^iv^	0.93	2.49	3.274 (7)	142
C3—H3···O3^iii^	0.93	2.50	3.352 (8)	153
C9—H9···O3^v^	0.93	2.48	3.391 (7)	168
C10—H10···O3	0.93	2.55	3.478 (7)	175
C12—H12···O4^ii^	0.93	2.58	3.423 (7)	151
C20—H20···O5^vi^	0.93	2.49	3.344 (8)	152
C8—H8···Cg2^ii^	0.93	3.07	3.638 (7)	113
C12—H12···Cg3^ii^	0.93	3.03	3.277 (7)	95
C23—H23···Cg1^vii^	0.93	2.61	3.509 (7)	164

Symmetry codes: (ii) *x*+1/2, −*y*+3/2, *z*+1/2; (iii) −*x*+3/2, *y*+1/2, −*z*+3/2; (iv) *x*−1/2, −*y*+3/2, *z*+1/2; (v) −*x*+2, −*y*+1, −*z*+1;  (vi) *x*−1, *y*, *z*−1; (vii) −*x*+1, −*y*+2, −*z*+1.
